# Prophylactic strategies to reduce cyclophosphamide-induced sterile hemorrhagic cystitis in dogs: a first-opinion practice retrospective study and a referral survey

**DOI:** 10.1093/jvimsj/aalag275

**Published:** 2026-10-26

**Authors:** Arturs Ivanovs, Christina MacKenzie, Zoe Wardle, Gina Pinchbeck, Heather Grieve, Peter-John Noble, David Roper Killick

**Affiliations:** Department of Small Animal Clinical Sciences, University of Liverpool, Neston, United Kingdom; Department of Small Animal Clinical Sciences, Institute of Infection, Veterinary and Ecological Sciences, University of Liverpool, Neston, United Kingdom; Department of Small Animal Clinical Sciences, Institute of Infection, Veterinary and Ecological Sciences, University of Liverpool, Neston, United Kingdom; Department of Small Animal Clinical Sciences, Institute of Infection, Veterinary and Ecological Sciences, University of Liverpool, Neston, United Kingdom; Department of Small Animal Clinical Sciences, Institute of Infection, Veterinary and Ecological Sciences, University of Liverpool, Neston, United Kingdom; School of Veterinary Medicine, University College Dublin, Dublin, Ireland; Department of Small Animal Clinical Sciences, Institute of Infection, Veterinary and Ecological Sciences, University of Liverpool, Neston, United Kingdom; Department of Small Animal Clinical Sciences, Institute of Infection, Veterinary and Ecological Sciences, University of Liverpool, Neston, United Kingdom

**Keywords:** cyclophosphamide, electronic health records, furosemide, metronomic chemotherapy, SAVSNET

## Abstract

**Background:**

Cyclophosphamide occasionally causes sterile hemorrhagic cystitis (SHC) in dogs. Prophylactic coadministration of furosemide reduces SHC risk, yet the extent of the implementation in practice is unknown.

**Hypothesis/Objectives:**

To explore the frequency of cyclophosphamide and furosemide coadministration in first opinion practice (FOP) and referral settings.

**Animals:**

Small Animal Surveillance Network (SAVSNET) searches identified 106 dogs receiving cyclophosphamide, of which 72 met the inclusion criteria.

**Methods:**

This study was composed of: (1) electronic health record (EHR) review using the SAVSNET database, in which data regarding dosage, co-prescribed medications, preventative measures, and suspected SHC occurrence were collated (2014-2024); and (2) a survey of referral oncology practitioners and educators (2023-2024) exploring contemporary approaches to SHC prevention.

**Results:**

Treatment approaches were evenly divided between maximum-tolerated dose cyclophosphamide (MTDC, 53%) and metronomic cyclophosphamide (MC, 47%). Furosemide was prescribed in 32% of cases, most commonly with MTDC protocols (53%). The overall frequency of suspected SHC was 15% (11/72); notably, no suspected SHC cases occurred in dogs receiving furosemide (*P* = .013). Survey results showed frequent use of prophylactic diuretics: 88% recommended furosemide for MTDC and 55% for MC protocols.

**Conclusions and clinical importance:**

Diuretic co-prescription with cyclophosphamide was inconsistent in FOP, which was unexpected given the prior evidence regarding reduction of risk of SHC. Greater standardization of guidance and improved dissemination of best-practice recommendations are necessary to optimize dog safety.

## Introduction

Cyclophosphamide is a commonly used chemotherapy agent in veterinary medicine. It is an alkylating agent commonly utilized in the treatment of lymphomas, carcinomas, and sarcomas in dogs,^1^ and forms part of multi-agent protocols such as COP and CHOP (cyclophosphamide, vincristine, doxorubicin, prednisolone). It is also used as a single agent or within metronomic protocols, in which low doses are administered continuously to target tumor angiogenesis and modulate the immune system.^2^^,^^3^ The broad range of indications and availability of intravenous and oral formulations have made it an attractive chemotherapy agent for use in first opinion practice (FOP).

After administration, cyclophosphamide undergoes hepatic activation to form several metabolites, including phosphoramide mustard, the primary antineoplastic compound capable of alkylating and cross-linking DNA strands, and acrolein, an inactive by-product responsible for sterile hemorrhagic cystitis (SHC).^4^^,^^5^

Reported incidence of SHC varies with dosing strategy from around 12.1% with high-dose cyclophosphamide to between 6.9% and 32% with metronomic protocols.^6^^,^^7^ Contrary to expectation, metronomic dosing does not reduce SHC risk,^1^^,^^6^ likely because of persistent bladder exposure to acrolein and cumulative dosing effects. The severity and duration are variable, and signs can last for weeks to months in severely affected dogs.

As there is no definitive treatment for SHC, prevention is considered essential. Strategies focus on reducing acrolein concentration and dwell time within the bladder. Prophylactic diuresis using the loop diuretic furosemide is recognized as one of the most effective measures,^1^^,^^6^^,^^7^ with evidence dating back to at least 2003 demonstrating reduced SHC incidence with concurrent administration.^8^ Additional studies subsequently report low or absent SHC frequencies using alternative cyclophosphamide administration strategies.^9^ Other recommended measures include increased fluid intake and frequent urination after treatment.

Although co-prescription of furosemide with cyclophosphamide is well established in referral oncology settings, its use in FOP is unknown and might be influenced by clinician awareness, training, and variation in practice protocols.

The purpose of this study was to describe the use of SHC preventative strategies, particularly furosemide co-prescription, in dogs receiving cyclophosphamide in FOP. Additionally, we surveyed referral clinicians and educators to evaluate current SHC prevention practices, recommendations provided to FOP practitioners, and undergraduate teaching on SHC prevention.

## Materials and methods

### Assessing anti-SHC prophylaxis in FOP using electronic health records

To evaluate approaches to SHC prophylaxis in FOP, we searched the Small Animal Veterinary Surveillance Network (SAVSNET) electronic health record (EHR) database using the SAVSNET Data Lab to identify cyclophosphamide prescriptions. Records from dogs seen in FOP between April 2014 and March 2024 were searched using the terms “cyclophosphamide” and “Endoxana.”

Cases were included if dogs received at least one dose of cyclophosphamide and had a subsequent appointment. Dogs were excluded if body weight data, dose information, or instructions for cyclophosphamide administration were missing, or if cyclophosphamide was discussed but never initiated.

Data extracted included demographics, cyclophosphamide dosing, co-prescribed medications, chemotherapy-induced adverse effects (including signs consistent with SHC), and prophylaxis strategies.

Dogs received various chemotherapy protocols. Protocols were classified as maximum tolerated (MTDC) or metronomic cyclophosphamide (MC), although some used modified dosing schedules. Dogs receiving pulse doses of 150 mg/m^2^ or greater were categorized as MTDC. Dogs receiving lower doses (<150 mg/m^2^) every 24-72 h for 5 days were classified as MC. Two MC subgroups were identified: low-intensity MC (>25 to < 150 mg/m^2^ every 2-3 days to weekly) and true-metronomic MC (<25 mg/m^2^ daily/every other day).

Suspected SHC was defined as gross or microscopic hematuria, with or without accompanying lower urinary tract signs,^10^ or an explicit SHC diagnosis recorded by the attending veterinary surgeon.

Prophylactic furosemide use, urinalysis, hematological monitoring, alternative medications, and owner education were recorded.

### Survey of practitioners working in clinical referral and educational contexts

As no multicenter referral EHR dataset reports SHC prophylaxis, and to explore the educational context of FOP findings, an online survey was conducted between October 2023 and July 2024. The survey explored prescribing practices, SHC prevention strategies, and how respondents advise FOP vets and teach undergraduate students. Responses regarding furosemide co-prescription were recorded using a 4-point Likert scale (“always,” “sometimes,” “rarely,” and “never”). For descriptive analysis, “always” and “sometimes” responses were grouped to represent routine prescribing behavior.

### Statistical analysis

Details of the statistical analyses are provided in the Appendix.

## Results

### Assessing anti-SHC prophylaxis in FOP using electronic health records

A total of 106 dogs with cyclophosphamide documented in their medical records between April 2014 and March 2024 were identified from the SAVSNET database. Thirty-four dogs were excluded. Reasons for exclusion, with some dogs meeting more than one exclusion criterion, including missing body weight data (*n* = 14), missing dose information (*n* = 12), missing administration instructions (*n* = 11), no subsequent appointment (*n* = 8), and cyclophosphamide being discussed but never initiated (*n* = 16). The remaining 72 dogs were included in the analysis.

Among the breeds represented, crossbreeds were most common (*n* = 18, 25%), followed by Labrador Retrievers (*n* = 10, 14%), Border Collies (*n* = 4, 6%), Cocker Spaniels (*n* = 4, 6%), and West Highland White Terriers (*n* = 3, 4%). The remaining breeds are listed in Table 1. Males were more prevalent (*n* = 42, 58%) than females (*n* = 30, 42%). Of the males, 24 (57%) were neutered, compared with 22 (73%) of the females. The mean age of dogs was 7.6 years (range: 0.4-14.6 years), and the mean body weight was 36.4 kg (range: 5.1-67.6 kg).

**Table 1:** Breed distribution of 72 dogs prescribed cyclophosphamide identified from EHRs.

Breed	Number of dogs
**Crossbreed**	18 (25%)
**Labrador Retrievers**	10 (14%)
**Border Collie**	4 (6%)
**Cocker Spaniel**	4 (6%)
**West Highland White Terrier**	3 (4%)
**Border Terrier, Boxer, Cockapoo, German Shepherd, Golden Retrievers, Jack Russel Terrier, Scottish Terrier, Springer Spaniel, Yorkshire Terrier**	2 each (3%)
**Basset Hound, Beagle, Briard, Cavalier King Charles Spaniel, Dogue de Bordeaux, Greyhound, Irish Setter, Miniature Schnauzer, Rottweiler, Shar-Pei, Shetland Sheepdog, Shih Tzu, Soft-coated Wheaten Terrier, Staffordshire Bull Terrier**	1 each (1%)

Abbreviation: EHR = electronic health record.

The most common indications for cyclophosphamide treatment were lymphoma (*n* = 25) and suspected lymphoma (*n* = 22). Other recorded indications included unspecified neoplasia (*n* = 12), multiple cutaneous masses (*n* = 3), mammary neoplasia (*n* = 2), suspected lymphoid leukemia (*n* = 1), mast cell tumor (*n* = 1), multiple rectal masses (*n* = 1), and a suspected sinus mass (*n* = 1).

Cyclophosphamide was prescribed more than once in 63 dogs (88%), and a single treatment was recorded in 9 dogs (13%). Medical records were reviewed to determine the cyclophosphamide dosing regimen used. The median prescribed dose was 150.2 mg/m^2^ (range: 9-298 mg/m^2^). Maximum-tolerated dose cyclophosphamide was prescribed in 38 dogs (53%), while 34 dogs (47%) received MC, including 30 dogs (42%) in the low-intensity subgroup and 4 dogs (6%) in the true-MC subgroup. Six dogs received cyclophosphamide IV as part of CHOP or high-dose COP protocols.

Prednisolone was the most frequently co-prescribed medication (*n* = 51, 71%), followed by prophylactic furosemide (*n* = 23, 32%). Concurrent prescription of prednisolone and furosemide was recorded in 12 dogs. One dog (1%) received meloxicam, while 8 dogs (11%) received cyclophosphamide alone.

Among the 23 dogs receiving furosemide, 4 (17%) received a single dose, IV, alongside cyclophosphamide, IV, although the exact dose was not available. The remaining 19 dogs (83%) received oral furosemide (tablet or liquid) with a mean dose of 1.36 mg/kg, PO (range: 0.5-2.3 mg/kg). The dosing regimens were recorded as follows: 8 dogs (42%) received a single dose on the day of MTDC; 6 dogs (32%) received split doses every 12 h; 1 dog (5%) received doses every 8 h; 2 dogs (11%) were treated once daily for 4 consecutive days; and 2 dogs (11%) received alternate-day dosing alongside MC therapy.

From the recorded dogs receiving MC, 3 (9%) were prescribed furosemide (*n* = 2 receiving low-intensity cyclophosphamide, and *n* = 1 receiving true-MC), 26 dogs (77%) received prednisolone (low-intensity subgroup only), 1 dog (3%) received meloxicam (low-intensity subgroup), and 5 (15%) received cyclophosphamide alone. In the MTDC group, 20 dogs (53%) received furosemide, 25 (66%) received prednisolone, and 3 (8%) had no co-prescription.

Preventative measures against SHC included furosemide (*n* = 23, 32%), pretreatment urinalysis (*n* = 20, 28%), and owner education (*n* = 22, 31%). None of the dogs receiving furosemide developed SHC, including the 12 dogs prescribed concurrent prednisolone. Sterile hemorrhagic cystitis was suspected in 11 of 72 dogs (15%; 95% CI, 8.8%-25.3%), all of which had not received furosemide. One of these occurred in a dog that received only a single dose of cyclophosphamide. There was an association between furosemide use and reduced odds of SHC (*P* = .013). In all cases, owners reported signs consistent with cystitis, and hematuria was confirmed by dipstick in 10 cases, with full urinalysis in 5. Six records indicated that urine culture was planned, although no results were available.

Pretreatment urinalysis was performed in 20 dogs (28%), with only 6 undergoing urinalyses before each treatment cycle. The majority of urinalyses were performed after the onset of clinical signs. Routine hematology was performed in 30 dogs (42%), and neutropenia was recorded in 4 (13%).

Owner education regarding SHC risk was documented in 22 cases (31%). Only 2 dogs that developed SHC had documented owner education (1 was receiving MTDC without co-prescription, and 1 was on MC with co-prescribed prednisolone). No association between owner education and SHC (*P* = .48) was detected.

Of the 11 dogs that developed SHC, 2 were receiving MTDC (mean dose per treatment 248.7 mg/m^2^), and 9 were receiving MC (mean dose per treatment 84.8 mg/m^2^), including 2 dogs on true-MC protocols (mean dose 16.5 mg/m^2^) and 7 on low-intensity regimens (mean 104.3 mg/m^2^). The overall mean cyclophosphamide dose among dogs that developed SHC was 114.5 mg/m^2^. Descriptive statistics of dogs with or without SHC are presented in Table 2. Treatment was discontinued in all affected dogs, with improvement documented in 8. One dog lacked follow-up, one continued to show signs for 40 days, and one was recorded as asymptomatic at the next visit despite incomplete documentation.

**Table 2:** Descriptive statistics of dogs with or without SHC incidence.

	SHC (*n* = 11)	No SHC (*n* = 61)
**Gender**		
**Males (*n* = 42, 58%)**	5 (7%)	37 (51%)
** Females (*n* = 30, 42%)**	6 (8%)	24 (33%)
**Age (years)**		
** Median**	7.54	7.62
** Range**	4.34-10.2	0.36-14.58
**Weight (kg)**		
** Median**	23.7	24
** Range**	7.45-22.05	5.6-67.6
**Furosemide (*n* = 23, 32%)**	0	23 (32%)
**No furosemide (*n* = 49, 68%)**	11 (15%)	38 (53%)
**Urinalysis (*n* = 20, 28%)**	6 (8%)	14 (19%)
**Owner education (*n* = 22, 31%)**	2 (3%)	20 (28%)
**MTDC (*n* = 38, 53%)**	2 (3%)	36 (50%)
** Mean dose per treatment (mg/m^2^)**	248.7	227.17
** Mean cumulative dose (mg/m^2^)**	*870.5*	*459.61*
**MC (*n* = 34, 47%)**	9 (13%)	25 (35%)
** Mean dose per treatment (mg/m^2^)**	84.77	55.16
** Mean cumulative dose (mg/m^2^)**	*1290.3*	*1328.88*
**Low-intensity subgroup (*n* = 30, 42%)**	7 (10%)	23 (32%)
** Mean dose per treatment (mg/m^2^)**	104.28	58.9
** Mean cumulative dose (mg/m^2^)**	*1407.42*	*1322.30*
**True-metronomic group (*n* = 4, 6%)**	2 (3%)	2 (3%)
** Mean dose per treatment (mg/m^2^)**	16.5	12
** Mean cumulative dose (mg/m^2^)**	*880.50*	*1404.5*

Percentages are calculated relative to the total number of dogs receiving cyclophosphamide (*n* = 72). Urinalysis included samples obtained before treatment or at the time of suspected urinary signs.

Abbreviations: MC = metronomic cyclophosphamide; MTDC = maximum tolerated dose cyclophosphamide; *n* = number of dogs in the specified group; SHC = sterile hemorrhagic cystitis.

An association was identified between cyclophosphamide protocol and SHC occurrence. Sterile hemorrhagic cystitis occurred in 2 of 38 dogs receiving MTDC (5%; 95% CI, 0.9%-17.3%) and 9 of 34 dogs receiving MC (27%; 95% CI, 13.9%-44.7%). Dogs receiving MC had higher odds of developing SHC than those receiving MTDC (OR = 6.48; 95% CI, 1.29-32.58; *P* = .02). Co-prescription of furosemide with MTDC was associated with reduced SHC occurrence (*P* = .008).

The median number of cyclophosphamide treatments administered before SHC onset was 16 (range: 1-185; mean 33). The median time to onset was 102 days (range: 14-353; mean 149 days). The median cumulative cyclophosphamide dose before SHC onset was 1000 mg/m^2^ (range: 96-2499 mg/m^2^; mean 1214 mg/m^2^). Two additional dogs showed cystitis-like symptoms, one had no follow-up available, and the other tested negative for hematuria but had discontinued treatment as a precaution. Another dog presented with hematuria but without other lower urinary tract signs and was euthanized before follow-up. An additional case involved hematuria with no further investigations performed because of lack of owner consent.

Between 2014 and 2024, the annual number of dogs treated with cyclophosphamide ranged from 1 to 12. Four of 12 dogs (33%) developed SHC in 2017, representing the highest annual proportion observed. In 2018 and 2019, SHC occurred in 2 of 10 (20%) and 2 of 9 (22%) dogs, respectively, with lower proportions recorded in subsequent years. The use of prophylactic furosemide increased from 1 of 12 dogs (8%) in 2017 to 4 of 10 (40%) in 2018 and 4 of 9 (44%) in 2019 and remained proportionally higher thereafter. Documented owner education was recorded in 6 of 12 dogs (50%) in 2017 and 4 of 10 (40%) in 2018. Annual proportions are presented in Figure 1.

**Figure 1:**
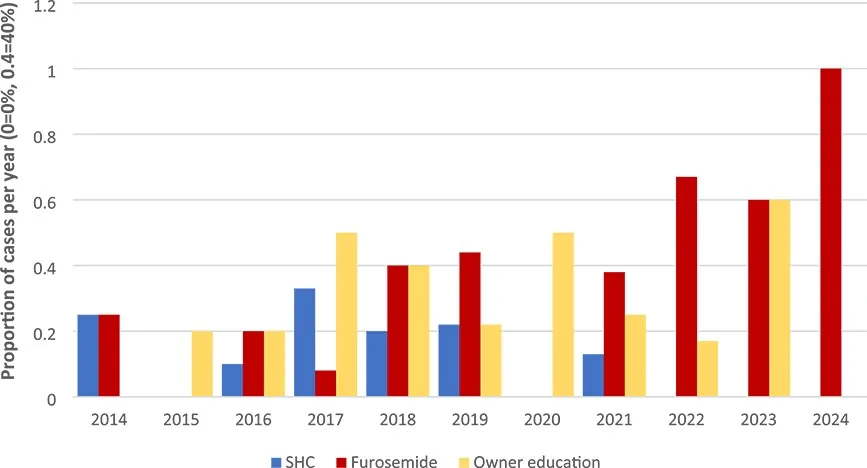
Annual proportions of cyclophosphamide-treated dogs that developed SHC, received prophylactic furosemide, or had documented owner education (2014-2024). Proportions were calculated relative to the total number of cyclophosphamide-treated dogs in each calendar year (range 1-12 dogs per year). Data for 2024 represent cases recorded between January and March 2024 (*n* = 1). Key SHC mitigation studies were published during this period.^1^^,^^9^^,^^10^^,^^14^^,^^19^ Abbreviation: SHC = sterile hemorrhagic cystitis.

### Survey of practitioners working in clinical referral and educational contexts

There were 86 respondents to the survey from 17 countries, including the United Kingdom (43%), France (8%), Germany (8%), Australia (8%), and others. Participants included 37 oncology diplomates (43%), 39 internal medicine diplomates (45%), and 10 SAMSoc (Small Animal Medicine Society) members (12%). As not all participants answered every question, response percentages were adjusted accordingly.

Among clinicians who regularly prescribe cyclophosphamide, 73 of 83 (88%) reported routinely co-prescribing furosemide with MTDC. For analysis, responses of “always” and “sometimes” were combined and categorized as “routine.” In comparison, 46 of 83 clinicians (55%) reported routine co-prescription with MC. Routine co-prescription of furosemide was therefore reported significantly more frequently when prescribing MTDC than MC (88% vs 55%; OR = 5.87; 95% CI, 2.66-12.94; *P* < .001). The full distribution of response categories (“always,” “sometimes,” “rarely,” and “never”) is presented in Figure 2.

**Figure 2:**
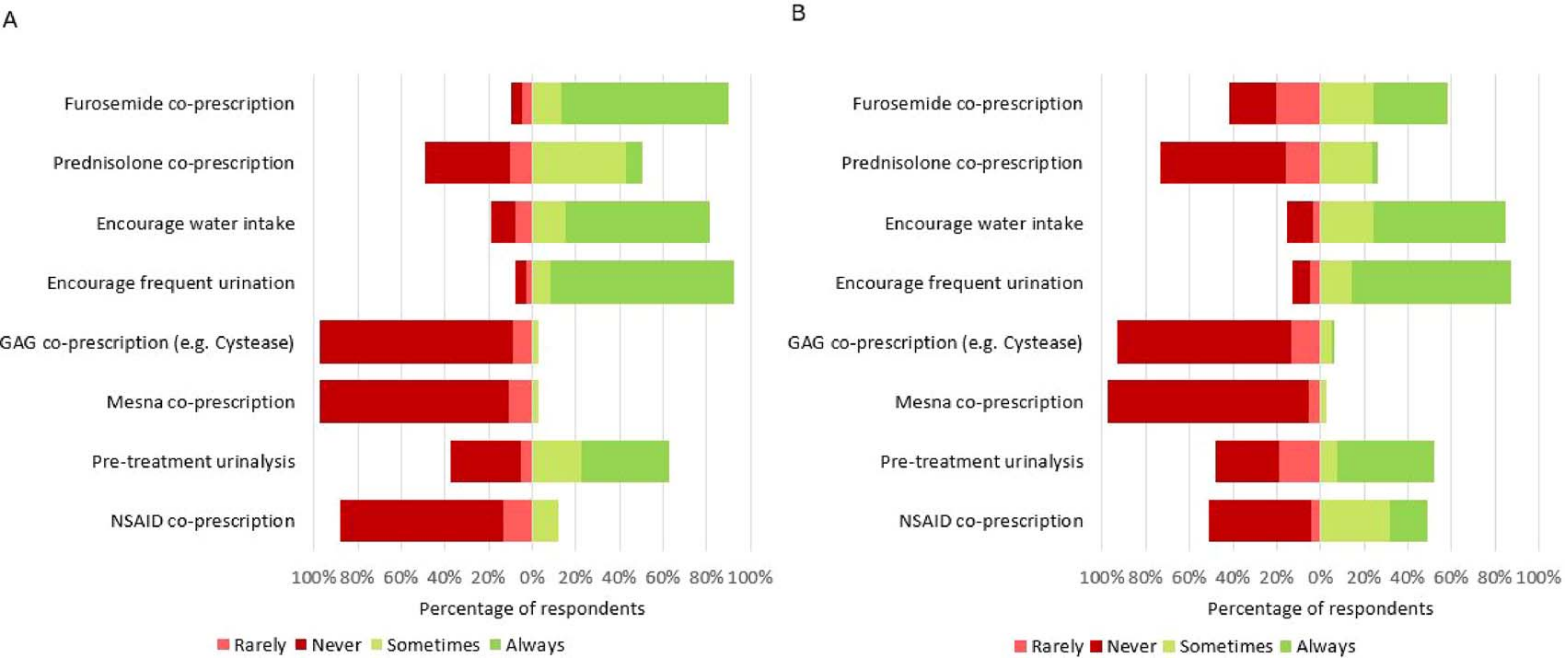
Frequency with which survey respondents reported using pharmacological and non-pharmacological prophylactic measures when prescribing (A) MTDC and (B) MC. Bars represent the proportion of respondents indicating “always,” “sometimes,” “rarely,” or “never” for each intervention. Interventions included pharmacological co-prescriptions (furosemide, prednisolone, NSAIDs, mesna, and GAGs) and behavioral recommendations (encouraging water intake and frequent urination). Abbreviations: GAGs = glycosaminoglycans; MC = metronomic cyclophosphamide; mesna = 2-mercaptoethane sulfonate sodium; MTDC = maximum tolerated dose cyclophosphamide; NSAIDs = nonsteroidal anti-inflammatory drugs.

Pretreatment urinalysis was also widely used when prescribing MTDC, with 50 clinicians (63%) reporting routine urinalysis before treatment initiation, compared with 41 clinicians (52%) when prescribing MC protocols.

Differences in prescribing patterns were also observed between specialists. Oncology diplomates reported more frequent “routine” co-prescription of furosemide than internal medicine diplomates when prescribing MC (62% vs 44%), although a statistical difference was not detected (possibly due to lack of statistical power; OR = 2.13; 95% CI, 0.85-5.33; *P* = .10). In contrast, routine use with MTDC was similar between the 2 groups (84% vs 85%; OR = 0.94; 95% CI, 0.28-3.20; *P* = .92). These differences are illustrated in Figure 3.

**Figure 3:**
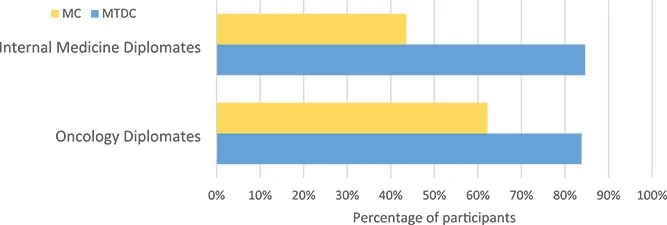
Comparison of routine furosemide co-prescription between oncology and internal medicine diplomates when prescribing MTDC and MC. Bars represent the proportion of respondents reporting routine use (combined “always” and “sometimes” responses). Abbreviations: MC = metronomic cyclophosphamide; MTDC = maximum tolerated dose cyclophosphamide.

When asked specifically about their current prescribing behaviors, 73 of 81 participants (90%) reported routinely prescribing furosemide with MTDC, whereas only 46 of 79 participants (58%) routinely used it with MC. Prednisolone co-prescription was less common overall, reported by 51% (39 of 77) for MTDC and 26% (20 of 76) for MC. Nonsteroidal anti-inflammatory drug co-prescription showed the reverse trend, with only 12% (11 of 76) reporting routine use alongside MTDC, compared with 49% (37 of 76) reporting routine use with MC. Nondrug preventative measures were widely recommended: increased water intake was encouraged by 81% for MTDC and 85% for MC, while increased opportunities to urinate were recommended by 93% and 87%, respectively.

Respondents also reported the teaching and advisory recommendations they provide to undergraduate students and first-opinion veterinarians. Recommendations were broadly aligned across groups. “Routine” recommendation of prophylactic furosemide was reported in 92% of MTDC scenarios and 76% of MC scenarios. “Routine” recommendations to increase water intake ranged from 78% to 96% for MTDC and 75%-91% for MC, while encouragement of more frequent urination ranged from 92% to 96% for MTDC, and 90%-96% for MC. Detailed numerical breakdowns by recipient group are presented in Table 3, and the combined teaching and advisory response distributions are illustrated in Figure 4.

**Table 3:** Routine recommendations (combined “always” and “sometimes” responses) provided in teaching and advisory settings to undergraduate students and first-opinion veterinarians.

Recommendation	Undergraduate teaching	Teaching first-opinion veterinary practitioners	Clinical advice to first-opinion veterinary practitioners
**Furosemide co-prescription**			
** MTDC**	21/23 (91%)	34/37 (92%)	55/60 (92%)
** MC**	17/23 (74%)	28/37 (76%)	43/60 (72%)
**Prednisolone co-prescription**			
** MTDC**	11/23 (48%)	18/35 (51%)	26/60 (43%)
** MC**	6/21 (29%)	11/35 (31%)	15/60 (25%)
**Encourage water intake**			
** MTDC**	21/22 (96%)	28/36 (78%)	49/60 (82%)
** MC**	20/22 (91%)	30/36 (83%)	45/60 (75%)
**Encourage frequent urination**			
** MTDC**	21/22 (96%)	34/36 (94%)	55/60 (92%)
** MC**	21/22 (96%)	34/36 (94%)	54/60 (90%)
**GAG co-prescription (eg, cystease)**			
** MTDC**	2/7 (29%)	3/35 (9%)	4/60 (7%)
** MC**	3/21 (14%)	2/35 (6%)	4/60 (7%)
**Mesna co-prescription**			
** MTDC**	3/21 (14%)	6/34 (18%)	6/60 (10%)
** MC**	1/21 (5%)	4/35 (11%)	4/60 (7%)
**Pretreatment urinalysis**			
** MTDC**	13/22 (59%)	20/35 (57%)	39/60 (65%)
** MC**	17/22 (77%)	24/35 (69%)	40/60 (67%)
**NSAID co-prescription**			
** MTDC**	1/22 (5%)	2/34 (6%)	6/60 (10%)
** MC**	11/22 (50%)	15/34 (44%)	20/60 (33%)

Abbreviations: MC = metronomic cyclophosphamide; mesna = 2-mercaptoethane sulfonate sodium; MTDC = maximum tolerated dose cyclophosphamide; NSAIDs = nonsteroidal anti-inflammatory drugs.

**Figure 4:**
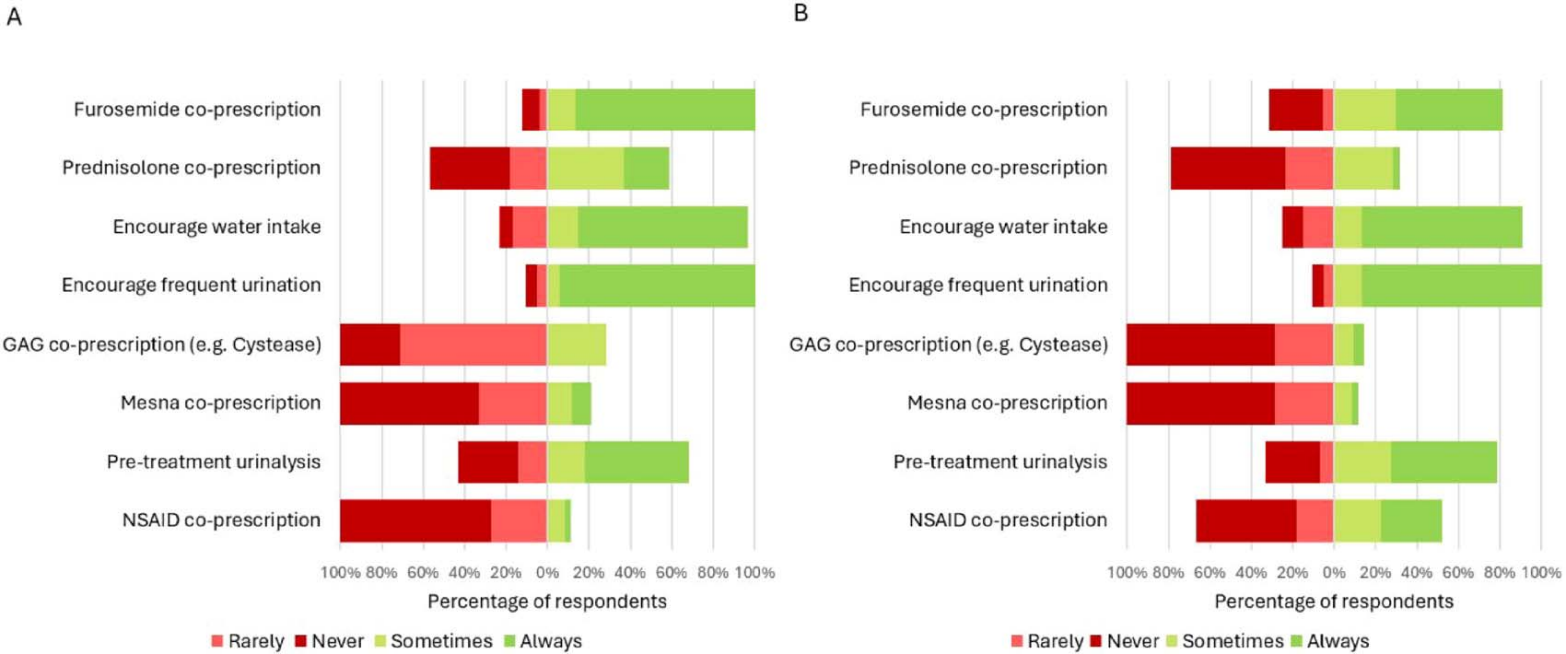
Frequency with which survey respondents teach or advise undergraduate students and first-opinion veterinarians on pharmacological and non-pharmacological prophylactic measures when prescribing (A) MTDC and (B) MC. Bars represent the proportion of respondents indicating “always,” “sometimes,” “rarely,” or “never” for each intervention. Interventions included pharmacological co-prescriptions (furosemide, prednisolone, NSAIDs, mesna, and GAGs) and behavioral recommendations (encouraging water intake and frequent urination). Abbreviations: GAGs = glycosaminoglycans; MC = metronomic cyclophosphamide; mesna = 2-mercaptoethane sulfonate sodium; MTDC = maximum tolerated dose cyclophosphamide; NSAIDs = nonsteroidal anti-inflammatory drugs.

Participants also rated their perception of furosemide’s benefit (*n* = 33). A considerable proportion expressed uncertainty about its utility in MC protocols: up to 30% (*n* = 10) considered furosemide rarely beneficial to non-beneficial when used with MC, compared with only 15% (*n* = 5) for MTDC. Specialty-based analysis demonstrated that oncology diplomates more commonly perceived furosemide as rarely beneficial with MC (24%, *n* = 8) compared with internal medicine diplomates (6%, *n* = 2), although the sample size is small and therefore this would need to be confirmed with a larger study. A similar pattern was observed for MTDC, although overall proportions were lower (12%, *n* = 4 vs 3%, *n* = 1, respectively).

Finally, respondents were asked to prioritize educational strategies to improve consistency in furosemide co-prescription. From 73 participants who answered this question, 56% (*n* = 41) ranked “include in formularies” as the most effective method. Verbal advice was the next most-preferred option (22%, *n* = 16), followed by undergraduate teaching (11%, *n* = 8), textbook inclusion (4%, *n* = 3), and examination content (1%, *n* = 1). A small proportion (6%, *n* = 4) felt that explicit educational reinforcement was not important.

## Discussion

Cyclophosphamide remains widely used in veterinary oncology; however, SHC is a clinically important adverse effect that can cause important and sometimes prolonged discomfort, leading to major reductions in quality of life of affected dogs. As no specific treatment for SHC exists, management is largely focused on control of clinical signs, making prevention the most appropriate approach. Several studies from referral populations report lower SHC incidence when prophylactic furosemide is administered concurrently with cyclophosphamide.^1^^,^^6^^,^^7^

Published research on SHC prophylaxis focuses on furosemide use in the referral setting. To evaluate the adoption of furosemide use in FOP, we utilized FOP EHRs. This approach was selected because survey responses can be affected by recall bias, a tendency to report idealized rather than actual behavior, or a combination of these factors.

The major finding of this study was the infrequent prescription of furosemide for SHC prophylaxis in FOP, with furosemide prescribed in only 32% of cases overall (53% in MTDC and 9% in MC protocols). Consistent with previous reports, no suspected SHC cases were identified among dogs prescribed furosemide. However, because this study was observational and protocol allocation was not randomized, this finding should be interpreted cautiously. The overall incidence of SHC among dogs treated with cyclophosphamide in the FOP setting was 15%, comparable to rates reported in referral populations when prophylactic diuretics were not routinely utilized, including 9%-15% in high-dose protocols and up to 32% in some metronomic cohorts.^6–8^^,^^11^ Although it might be assumed that SHC incidence would be lower in FOP due to shorter travel times and increased opportunity for owner monitoring, this study did not support that assumption.

There is a substantial reduction in SHC incidence with prophylactic furosemide, including decreases from 9% to 1.2% and from 12.1% to 2.46% in high-dose protocols, with similar reductions reported in metronomic regimens.^1^^,^^6^^,^^8^^,^^12^ In the present cohort, none of the dogs receiving furosemide developed SHC, although the limited sample size necessitates cautious interpretation of this observation. This finding should be considered hypothesis-generating rather than confirmatory. The mean prophylactic dose of furosemide (1.36 mg/kg; range 0.5-2.3 mg/kg) was consistent with previously published recommendations.^8^^,^^12^ However, prescribing schedules varied substantially. Only 2 MTDC cases received furosemide in accordance with the BSAVA (2023)^13^ guidance, recommending 4 doses at 12-h intervals, while most received a single bolus dose. Although induction of diuresis is desirable, possible adverse effects, including dehydration, hyponatremia, and nephrotoxicity, must be considered.^4^^,^^14^^,^^15^ These findings show variability in implementation within FOP and emphasize the need for clearer, standardized guidance for general practice.

Cyclophosphamide protocols in FOP were more heterogeneous than those typically described in referral settings, potentially reflecting variation in clinician preference, case characteristics, local protocols, or other factors. This variation allowed exploratory evaluation of dose and frequency effects. Interestingly, an association was observed between dosing intensity and SHC development, with the majority of cases occurring in dogs receiving moderate dose, moderate frequency regimens, such as those commonly used in low-dose COP protocols. This finding suggests that cumulative bladder exposure might be influenced not only by the total dose but also by the administration schedule. Although differences in SHC risk were observed between MTDC and MC regimens overall, no statistical difference was identified between low-intensity and true-metronomic subgroups, possibly reflecting limited statistical power.

Diagnosis of SHC ideally requires documented hematuria in the absence of bacterial infection and, in some studies, ultrasonographic exclusion of alternative causes. Strict diagnostic criteria exclude dogs that do not meet all requirements, whereas other criteria require ultrasonographic confirmation in addition to negative urine culture results.^6^^,^^12^ In contrast, as this was a retrospective FOP study relying on EHRs, urine culture data were frequently unavailable, and SHC diagnosis was based on documented hematuria and compatible clinical signs. Other lower urinary tract conditions, including bacterial cystitis or urolithiasis, can present identically,^16^ and some degree of misclassification is therefore possible. Therefore, the diagnosis of SHC in this study should be considered presumptive rather than definitive. Similarly, pragmatic case definitions are employed in other retrospective analyses; for example, SHC is defined as clinical signs that resolve after discontinuation of cyclophosphamide.^1^ Although the broader case definition used in the present study carries a risk of overestimation, inclusion of such borderline cases would have only marginally altered the overall incidence. This limitation reflects the built-in constraints of retrospective EHR-based studies in the first opinion practice, where diagnostic work-up and documentation are variable.

Owner education and urinalysis have been proposed as additional mitigation strategies.^6^^,^^17^ In this study, no association was observed between documented owner education and SHC. However, documentation practices can vary substantially between clinicians and practices, particularly for verbal advice, and urinalysis was inconsistently recorded (fewer than 30% of cases). Most urinalyses were performed reactively after clinical signs rather than as routine screening. Given the low frequency of pretreatment monitoring and the risk of detecting bias, statistical assessment of urinalysis as a preventive strategy was not undertaken. Additionally, some normal urinalysis might have been performed, but not recorded. Consequently, the true impact of routine screening on early detection or prevention of SHC could not be reliably evaluated. These findings should therefore be considered exploratory rather than conclusive and might support a need for easily accessible standardized educational material, such as written instruction sheets or online guidance.

Temporal analysis demonstrated the highest annual SHC proportion in 2017, followed by increased furosemide co-prescription and improved documentation of owner education in subsequent years. Although SHC proportions were lower in later years, the small annual case numbers and retrospective design preclude conclusions regarding causality or true temporal change. Data for 2024 represented only a partial year (January—March), during which only one dog received cyclophosphamide; therefore, proportions for that year should be interpreted cautiously. The timing of multiple key publications addressing SHC mitigation^1^^,^^6^^,^^12^^,^^17^ might have helped to increase awareness; however, this association remains speculative.

Given the apparent evidence-practice gap identified in FOP regarding furosemide use for SHC prophylaxis, a survey of referral clinicians was undertaken to explore prescribing practice, advice, and education within the referral setting. As referral EHRs were not available at scale, clinical practice questions were included in the survey, acknowledging the possibility of self-reported data idealization.

Survey respondents reported substantially higher routine furosemide use than observed in SAVSNET data. Oncology diplomates reported routine co-prescription in 84% of MTDC cases and 62% of MC cases, while internal medicine diplomates reported 85% and 44%, respectively. Furosemide was ranked as the most important preventative measure, particularly in MTDC protocols. These findings suggest broad agreement among specialists regarding the routine use of prophylaxis in MTDC regimens. However, it is important to recognize that the survey, conducted between October 2023 and July 2024 reflects contemporary self-reported behavior and does not capture changes over time in the same manner as the longitudinal FOP dataset. It is also the case that metronomic chemotherapy was not defined in the survey, and consequently, it might be that responses pertaining to metronomic chemotherapy in the survey map best to “true-MC” cases in the FOP data. Nonetheless, reported routine prophylaxis in the referral setting was higher than that observed in FOP data, even during the later study period.

Greater variability was observed in the prophylactic approaches used with metronomic cyclophosphamide. Among respondents who rated perceived benefit (*n* = 33), uncertainty regarding the utility of furosemide was more pronounced in the MC setting than in the MTDC protocols. Approximately one-third considered furosemide rarely beneficial or not beneficial in MC regimens, compared with a substantially smaller proportion for MTDC. This might reflect differences in perceived risk profile between the 2 approaches. Because metronomic cyclophosphamide is administered at lower, continuous doses, clinicians might perceive the risk of sterile hemorrhagic cystitis to be reduced relative to maximum tolerated dose protocols, possibly influencing views on the necessity of prophylactic diuresis.

Specialty-based variation was also evident, with oncology diplomates more often rating furosemide as rarely beneficial in MC than internal medicine diplomates. However, a numeric difference in routine co-prescription of furosemide between the 2 specialist groups, although close, did not reach the threshold for statistical significance. This disparity might reflect differences in the prescribing frequency and cumulative clinical exposure to metronomic protocols. Although subgroup sizes were modest and conclusions exploratory, these findings indicate that uncertainty regarding optimal prophylaxis in MC protocols persists even within referral settings.

The disparity between referral and FOP findings emphasizes the importance of effective knowledge translation. While specialist consensus and updated guidelines (eg, BSAVA 2023) can promote standardization within referral practice, dissemination and uptake of these recommendations in FOP settings might be influenced by educational exposure, local protocols, resource restrictions, and perceived risk–benefit considerations. The persistence of lower prophylactic uptake in FOP despite current referral consensus highlights an ongoing evidence-practice gap and identifies a clear target for improved clinical guidance.

Several limitations warrant consideration. First, the FOP component was designed retrospectively and relied on the completeness and accuracy of EHRs. Excluding 34 of 106 (32%) identified cases could introduce selection bias. As it was not possible to determine whether the excluded cases differed from those included, the direction and magnitude of any potential selection bias cannot be determined from the available data. It also describes UK veterinary practice, and it is unknown if the situation is different in other countries. Sterile hemorrhagic cystitis diagnosis was based on recorded clinical signs and hematuria, with incomplete culture confirmation, and some degree of misclassification is therefore possible. Prescription records do not confirm medication administration or owner compliance; thus, documented furosemide prescriptions might overestimate actual prophylactic exposure. The heterogeneity of cyclophosphamide protocols in the first opinion practice might also have influenced both SHC risk and prescribing decisions. Information regarding compounded vs commercially manufactured cyclophosphamide formulation was not consistently available and therefore could not be assessed as a potential contributor to SHC risk. This is relevant given previously reported variability in the potency and stability of compounded chemotherapy formulations.^18^^,^^19^ The absence of cases in 2020 likely reflects reduced clinical activity during the COVID-19 pandemic rather than a true absence of SHC. Finally, the cross-sectional nature of the referral survey limits inference regarding historical changes in specialist practice.

## Conclusion

In summary, despite strong evidence supporting its protective effect, furosemide was underutilized in FOP, representing the principal finding of this study. The frequency of SHC observed in FOP was comparable to that reported in previous retrospective studies from referral settings where prophylactic diuretics were not routinely used. Although several preventive measures were inconsistently used, furosemide co-prescription appeared to increase over time, while SHC occurrence declined. This temporal pattern might reflect increasing awareness and improved implementation of risk-reduction strategies in general practice; however, these observations should be interpreted cautiously given the limited data available.

The complementary survey of specialists highlighted a markedly higher and more consistent use of prophylactic strategies, particularly the co-prescription of furosemide in MTDC protocols, while opinions regarding MC prophylaxis remain more variable. This difference between specialist recommendations and real-world FOP underscores a persistent evidence-practice gap.

Together, these findings emphasize the need for clearer, standardized guidelines on SHC prevention, improved dissemination of best-practice recommendations and more structured client education to support early recognition and risk education.

## Supplementary Material

Supplementary_Material_Survey_aalag275

## Data Availability

Data available upon reasonable request to corresponding author. Restricted due to SAVSNET policies.

## Off-label antimicrobial declaration

The authors declare no off-label use of antimicrobials.

## Institutional animal care and use committee or other approval declaration

SAVSNET operates under ethical approval from the University of Liverpool Ethics Committee (RETH001081), and access to the data for this study was granted following application to the SAVSNET Data Access and Publication Panel.

## Appendix

### Materials and methods

This study evaluated practice in first opinion practice (FOP), clinical referral, and educational contexts.

#### Assessing anti-SHC prophylaxis in FOP using electronic health records

Small Animal Surveillance Network (SAVSNET) recruits by convenience from first opinion veterinary practices across the United Kingdom that are using compatible practice management software. The electronic health record (EHR) data are then collected in near real-time. The SAVSNET EHR dataset includes information about the animal (eg, species, breed, sex, neuter status, age, owner’s postcode, insurance, and microchipping status), as well as de-identified free text clinical narratives (CNs) and prescribed treatments. Small Animal Surveillance Network data collection has been more completely described previously.^20^

Identified medical records were reviewed for inclusion by the authors. Cases were included when all of the following criteria were met:

1. Patient was a dog.
2. Dog was prescribed at least one dose of cyclophosphamide.
3. Dog had at least one appointment subsequent to cyclophosphamide prescription.

Dogs receiving a single dose of cyclophosphamide were retained in the analysis, as sterile hemorrhagic cystitis (SHC) has previously been reported after a single administration.^8^^,^^21^

Dog body weight was often recorded inconsistently or infrequently; therefore, the weight recorded closest to the first cyclophosphamide dose was used to calculate body surface area (BSA), according to BSAVA guidelines (2023).^13^

Where possible, the nature of the protocol was determined either: maximum tolerated (as part of the COP or CHOP protocol) or metronomic cyclophosphamide regimen; however, if cyclophosphamide dosing changed during the treatment, the highest recorded dose was used in analysis.

For the purposes of this part of the study, SHC was defined as the presence of gross or microscopic hematuria plus clinical signs of lower urinary tract disease, or an explicit diagnosis of SHC recorded by the attending veterinary surgeon.

For dogs diagnosed with SHC, the interval between first cyclophosphamide administration and onset of urinary signs was recorded. Data regarding any mitigation strategies, including the route, frequency, and dose of prophylactic furosemide, were also recorded. The frequency of urinalysis and hematological monitoring was documented, and alternative medications were noted.

Clinical notes were reviewed for documentation indicating that owners had been educated about the risk of SHC, including hydration advice, increased frequency of urination, or written information provided.

#### Survey of practitioners working in clinical referral and educational contexts

Using this tool, we assessed contemporary prescribing practices and SHC prevention strategies among oncology and internal medicine diplomates, referral practitioners, and key opinion leaders. We also explored how they advise and teach FOP to vets and undergraduate students. A copy of the survey is available in the Supplementary material.

The survey was distributed with assistance from the European College of Veterinary Internal Medicine (ECVIM-CA) and the Small Animal Medicine Society (SAMSoc). The questionnaire was hosted on Microsoft Forms.

### Statistical analysis

Descriptive and comparative statistical analyses were performed. Associations between categorical variables and SHC development were assessed using chi-square tests, or 2-sided Fisher’s exact test for comparison involving sparse data or zero-event cells. Effect sizes for categorical comparisons were reported as odds ratios (ORs) with 95% CIs. Confidence intervals for proportions were calculated using the Wilson score method. A *P*-value < .05 was considered statistically significant. Statistical analyses were performed using GraphPad Prism and MedCalc. No adjustments for multiple comparisons were applied due to the exploratory nature of the study and the small sample size.

## Abbreviations

CHOP: cyclophosphamide, hydroxydaunorubicin (doxorubicin), oncovin (vincristine), prednisolone
COP: cyclophosphamide, oncovin (vincristine), prednisolone
EHR: electronic health record
FOP: first opinion practice
MC: metronomic cyclophosphamide
MTDC: maximum tolerated dose cyclophosphamide
OR: odds ratio
SAVSNET: Small Animal Surveillance Network
SHC: sterile hemorrhagic cystitis
