# Supplementary material for: Prophylactic strategies to reduce cyclophosphamide-induced sterile hemorrhagic cystitis in dogs: a first-opinion practice retrospective study and a referral survey
Source: J Vet Intern Med. 2026 Oct 26;40(5):aalag275. doi: 10.1093/jvimsj/aalag275 (PMC13706619; doi:10.1093/jvimsj/aalag275)
Supplement: Supplementary file 1 [file supplementary_material_survey_aalag275.pdf]

What steps do you take and advise to prevent sterile haemorrhagic cystitis after cyclophosphamide?

1. *

Please select 2 options.

- [ ] I confirm that I have read the information sheet for the above study. I have had the opportunity to consider the information. I agree that the anonymous data I provide will be used for research purposes. I consent to take part in this study.
- [ ] I understand that my participation is voluntary and that I am free to withdraw from the survey prior to its submission without giving any reason.

About you

2.Please state the country in which you are working *

3.What proportion of your clinical time is spent in each of these settings? *

	100%	75 - 99%	50 - 75%	25 - 50%	Up to 25%	I do not work in this setting
Referral oncology	○	○	○	○	○	○
Referral other disciplines	○	○	○	○	○	○
First opinion practice	○	○	○	○	○	○

4.What is your highest level of clinical qualification / endeavour in each of the following areas? *

	ECVIM / ACVIM Diploma	Studying for Diploma	Postgraduate certificate or Masters	Studying for certificate or Masters	None
Oncology	○	○	○	○	○
Internal medicine	○	○	○	○	○
Cardiology	○	○	○	○	○
Other clinical discipline	○	○	○	○	○

Measures to prevent sterile haemorrhagic cystitis (SHC) in your clinical cases

5.I have prescribed cyclophosphamide treatment for DOGS with cancer *

- ◯ Yes
- ◯ No

6.When prescribing MAXIMUM TOLERATED DOSE cyclophosphamide (e.g. in COP or CHOP protocols) to DOGS in order to reduce the risk SHC I prescribe / advise:

	Always	Sometimes	Rarely	Never
Furosemide co-prescription	◯	◯	◯	◯
Prednisolone co-prescription	◯	◯	◯	◯
Increasing water intake	◯	◯	◯	◯
Increasing oppor tunities to urinate	◯	◯	◯	◯
Cystease or other GAG co-prescription	◯	◯	◯	◯
Mesna co-prescription	◯	◯	◯	◯
Pre-treatment urinalysis (if free catch available)	◯	◯	◯	◯
NSAID co-prescription	◯	◯	◯	◯

7. When prescribing METRONOMIC cyclophosphamide to DOGS in order to reduce the risk of SHC I prescribe / advise:

	Always	Sometimes	Rarely	Never
Furosemide co-prescription	○	○	○	○
Prednisolone co-prescription	○	○	○	○
Increasing water intake	○	○	○	○
Increasing oppor tunities to urinate	○	○	○	○
Cystease or other GAG co-prescription	○	○	○	○
Mesna co-prescription	○	○	○	○
Pre-treatment urinalysis (if free catch available)	○	○	○	○
NSAID co-prescription	○	○	○	○

8. Please select the circumstances (if any) that would make you MORE likely to prescribe furosemide/prednisolone/GAGs/Mesna/NSAIDs to a dog that is to be treated with cyclophosphamide.

- ☐ The patient is male
- ☐ The patient is female
- ☐ The patient is <6months old
- ☐ The patient is considered to be senior
- ☐ The patient is underweight
- ☐ The patient is overweight
- ☐ The patient has undergone a different cancer treatment within the last 6 months
- ☐ The patient suffers from an immune mediated disease
- ☐ The patient suffers from a hepatic disease
- ☐ The patient suffers from diabetes mellitus
- ☐ The cyclophosphamide is to be administered intravenously
- ☐ The cyclophosphamide is to be administered orally
- ☐ Other

Cyclophosphamide induced SHC prevention education and advice

9.I teach undergraduates oncology *

- ◯ Yes
- ◯ No

10.When teaching **undergraduates** about prescribing MAXIMUM TOLERATED DOSE cyclophosphamide (e.g. within COP or CHOP protocols) to DOGS in order to reduce risk of SHC I teach the following:

	Always	Sometimes	Rarely	Never
Furosemide co-prescription	◯	◯	◯	◯
Prednisolone co-prescription	◯	◯	◯	◯
Increasing water intake	◯	◯	◯	◯
Increasing oppor tunities to urinate	◯	◯	◯	◯
Cystease or other GAG co-prescription	◯	◯	◯	◯
Mesna co-prescription	◯	◯	◯	◯
Pre-treatment urinalysis (if free catch available)	◯	◯	◯	◯
NSAID co-prescription	◯	◯	◯	◯

11. When teaching **undergraduates** about prescribing METRONOMIC cyclophosphamide to DOGS in order to reduce risk of SHC I teach the following:

	Always	Sometimes	Rarely	Never
Furosemide co-prescription	◯	◯	◯	◯
Prednisolone co-prescription	◯	◯	◯	◯
Increasing water intake	◯	◯	◯	◯
Increasing oppor tunities to urinate	◯	◯	◯	◯
Cystease or other GAG co-prescription	◯	◯	◯	◯
Mesna co-prescription	◯	◯	◯	◯
Pre-treatment urinalysis (if free catch available)	◯	◯	◯	◯
NSAID co-prescription	◯	◯	◯	◯

12. I teach qualified first opinion vets in oncology (e.g. CPD, certificate courses) *

◯ Yes

◯ No

13.When teaching **first opinion vets** about prescribing MAXIMUM TOLERATED DOSE cyclophosphamide (e.g. within COP or CHOP protocols) to DOGS in order to reduce risk of SHC I teach the following:

	Always	Sometimes	Rarely	Never
Furosemide co-prescription	◯	◯	◯	◯
Prednisolone co-prescription	◯	◯	◯	◯
Increasing water intake	◯	◯	◯	◯
Increasing oppor tunities to urinate	◯	◯	◯	◯
Cystease or other GAG co-prescription	◯	◯	◯	◯
Mesna co-prescription	◯	◯	◯	◯
Pre-treatment urinalysis (if free catch available)	◯	◯	◯	◯
NSAID co-prescription	◯	◯	◯	◯

14.When teaching **first opinion vets** about prescribing METRONOMIC cyclophosphamide to DOGS in order to reduce risk of SHC I teach the following:

	Always	Sometimes	Rarely	Never
Furosemide co-prescription	◯	◯	◯	◯
Prednisolone co-prescription	◯	◯	◯	◯
Increasing water intake	◯	◯	◯	◯
Increasing oppor tunities to urinate	◯	◯	◯	◯
Cystease or other GAG co-prescription	◯	◯	◯	◯
Mesna co-prescription	◯	◯	◯	◯
Pre-treatment urinalysis (if free catch available)	◯	◯	◯	◯
NSAID co-prescription	◯	◯	◯	◯

15. I provide clinical oncology case advice for veterinarians in practice *

◯ Yes

◯ No

16. When advising **veterinary surgeons on clinical cases** requiring prescription of MAXIMUM TOLERATED DOSE cyclophosphamide (e.g within COP or CHOP protocols) to DOGS in order to reduce the risk of SHC I advise the following: *

	Always	Sometimes	Rarely	Never
Furosemide co-prescription	◯	◯	◯	◯
Prednisolone co-prescription	◯	◯	◯	◯
Increasing water intake	◯	◯	◯	◯
Increasing oppor tunities to urinate	◯	◯	◯	◯
Cystease or other GAG co-prescription	◯	◯	◯	◯
Mesna co-prescription	◯	◯	◯	◯
Pre-treatment urinalysis (if free catch available)	◯	◯	◯	◯
NSAID co-prescription	◯	◯	◯	◯

17. When advising **veterinary surgeons on clinical cases** requiring prescription of METRONOMIC cyclophosphamide to DOGS in order to reduce the risk of SHC I advise the following: *

	Always	Sometimes	Rarely	Never
Furosemide co-prescription	○	○	○	○
Prednisolone co-prescription	○	○	○	○
Increasing water intake	○	○	○	○
Increasing oppor tunities to urinate	○	○	○	○
Cystease or other GAG co-prescription	○	○	○	○
Mesna co-prescription	○	○	○	○
Pre-treatment urinalysis (if free catch available)	○	○	○	○
NSAID co-prescription	○	○	○	○

18. If you rarely or never advise vets to co-prescribe furosemide with cyclophosphamide (in order to reduce the risk of sterile haemorrhagic cystitis) which of the following best describes why? (multiple answers allowed)

- ☐ Plenty of evidence therefore qualified vets should know already
- ☐ It does not tend to come up in the discussion
- ☐ Advise tends to be at a more general level (e.g. which protocol to use)
- ☐ I don't find furosemide is beneficial in cases receiving maximum tolerated dose cyclophosphamide
- ☐ I don't find furosemide is beneficial in cases receiving metronomic cyclophosphamide
- ☐ Cost
- ☐ My advice focuses on more important aspects of the case
- ☐ I always or sometimes advise use of furosemide in these cases
- ☐ Other

19. Please rank each of the following as ways to teach veterinarians to co-prescribe furosemide with cyclophosphamide? (drag to preferred order)

- Verbally (such as in conferences/lectures)
- Undergraduate lecture notes
- Include in formularies (e.g. BSAVA Formulary and Plumb)
- Include in textbooks
- Include in examinations
- I do not consider it important to co-prescribe furosemide with cyclophosphamide

20. If there is another medication or approach you use or advise to reduce the risk of cyclophosphamide induced sterile haemorrhagic cystitis, that is not mentioned above then please describe it in the box below.
This box can also be used for any comments you would like to make.
